# Measuring racial and ethnic disparities in traffic enforcement with large-scale telematics data

**DOI:** 10.1093/pnasnexus/pgac144

**Published:** 2022-07-30

**Authors:** William Cai, Johann Gaebler, Justin Kaashoek, Lisa Pinals, Samuel Madden, Sharad Goel

**Affiliations:** Department of Management Science and Engineering, Stanford University, Stanford, CA 94305, USA; Institute for Computational and Mathematical Engineering, Stanford University, Stanford, CA 94305, USA; Cambridge Mobile Telematics Inc., Cambridge, MA 02142, USA; Cambridge Mobile Telematics Inc., Cambridge, MA 02142, USA; Cambridge Mobile Telematics Inc., Cambridge, MA 02142, USA; Harvard Kennedy School, Cambridge, MA 02138, USA

**Keywords:** discrimination, disparate impact, policing, telematics

## Abstract

Past studies have found that racial and ethnic minorities are more likely than White drivers to be pulled over by the police for alleged traffic infractions, including a combination of speeding and equipment violations. It has been difficult, though, to measure the extent to which these disparities stem from discriminatory enforcement rather than from differences in offense rates. Here, in the context of speeding enforcement, we address this challenge by leveraging a novel source of *telematics* data, which include second-by-second driving speed for hundreds of thousands of individuals in 10 major cities across the United States. We find that time spent speeding is approximately uncorrelated with neighborhood demographics, yet, in several cities, officers focused speeding enforcement in small, demographically nonrepresentative areas. In some cities, speeding enforcement was concentrated in predominantly non-White neighborhoods, while, in others, enforcement was concentrated in predominately White neighborhoods. Averaging across the 10 cities we examined, and adjusting for observed speeding behavior, we find that speeding enforcement was moderately more concentrated in non-White neighborhoods. Our results show that current enforcement practices can lead to inequities across race and ethnicity.

Significance Statement There is widespread concern that traffic stops—the most common form of police–civilian interaction—suffer from racial bias. It is often difficult, though, to rigorously assess the extent to which disparate policing patterns are driven by discrimination as opposed to differences in violation rates by group. Here, we use a novel source of telematics data to directly compare speeding enforcement with the true underlying frequency of speeding. We find that while the rate of speeding is approximately uncorrelated with neighborhood demographics, enforcement is often concentrated in relatively small, demographically nonrepresentative neighborhoods. Our approach lets us address long-standing statistical challenges in discrimination research, and illustrates the value of modern data collection methods for informing pressing policy problems.

## Introduction

Traffic violations are the most common cause of police–civilian contact, with tens of thousands of stops occurring every day in the United States (1). The ubiquity of traffic stops, in addition to the fines, loss of dignity, and other burdens they place on stopped civilians—particularly people of color—have made them an important object of study in discrimination research (2–16). However, the lack of a clear benchmark (17) poses a fundamental challenge in quantifying racial and ethnic disparities in policing patterns: traffic violations that do not result in police action are typically not recorded, making it difficult to disentangle the role of differences in driving behavior from differences in police enforcement in creating disparities (18,19).

To overcome this challenge, our analysis uses vehicle telematics data, which include vehicle location and speed over time, collected from drivers nationwide. With these data, we estimate the true underlying rate of speeding violations by geographic area, mitigating the benchmark problem that has stymied past efforts. To complete our analysis, we combined aggregated telematics data from tens of millions of trips with large-scale public records on speeding enforcement drawn from nearly 800,000 speeding stops in 10 American cities to compare speeding enforcement across neighborhoods with different demographic compositions, after adjusting for driving behavior.

**Fig. 1.**
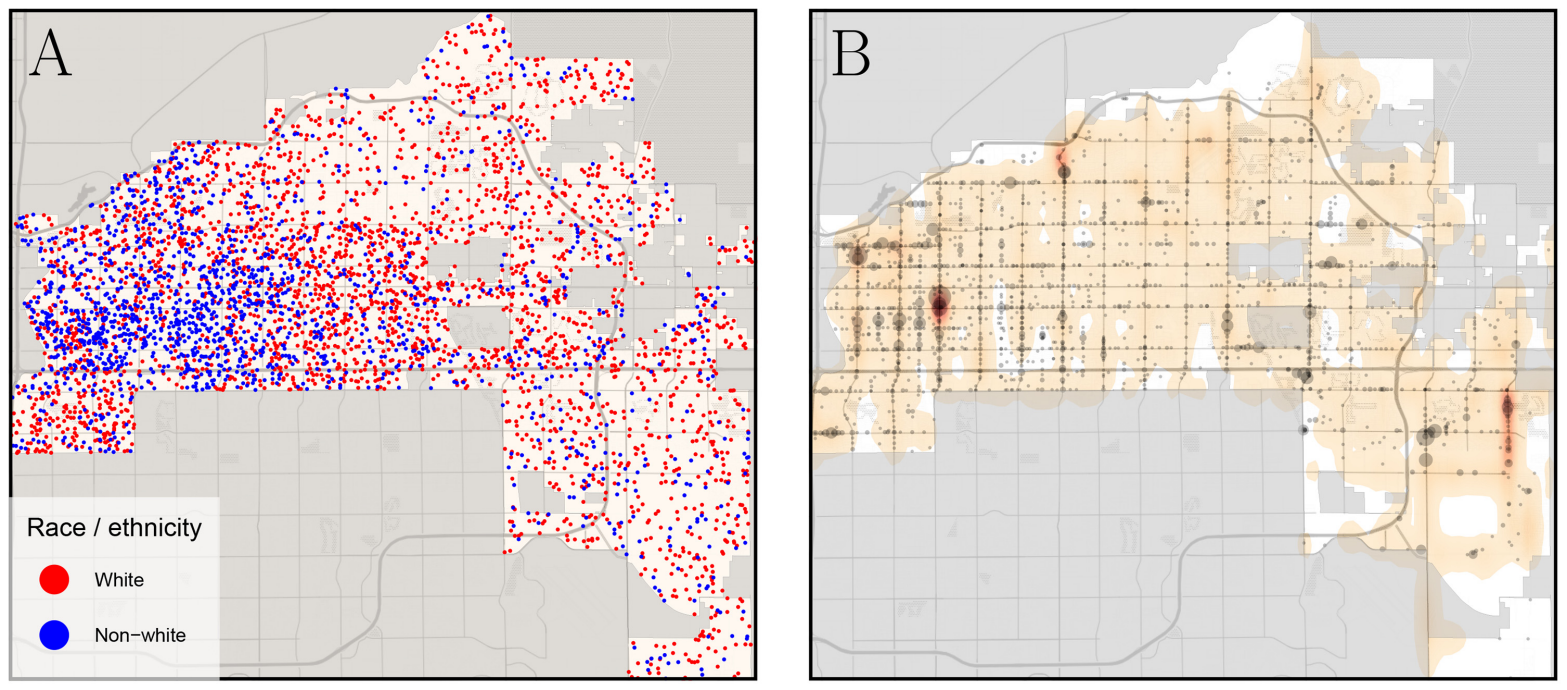
For Mesa, Arizona, a comparison of the city’s demographic composition, the location of speeding events, as measured by telematics data, and the location of speeding stops. (A) The demographics of residents, where red points represent non-Hispanic White residents and blue points represent non-White residents. (B) The density of speeding stops recorded by police officers, indicated by gray dots, where the area of the points is proportional to the number of stops at a single location; stops for speeding are overlaid with a heatmap of speeding events, as estimated from telematics data. Comparing the two panels, we find higher concentrations of recorded speeding stops in neighborhoods, which have high proportions of residents of color, despite that fact that actual speeding events are more evenly distributed across the city. Thus, in Mesa, it appears that speeding is more heavily enforced in predominately minority neighborhoods. We note that this pattern does not hold in every city we study, and, in some locations, speeding is disproportionately enforced in predominately White neighborhoods.

To illustrate the benchmark problem, and our approach to addressing it, we first consider Mesa, Arizona. Like many American cities (20), Mesa exhibits patterns of residential segregation, with the city’s racial and ethnic minorities concentrated in the northwest neighborhoods, as shown in Fig. 1(A). Using data from the Open Policing Project (21), we plot, in Fig. 1(B), speeding stops recorded by the Mesa Police Department between 2014 and 2016, where the area of the gray circles indicates the number of stops at that location. Visually inspecting the map, we see that speeding stops are concentrated in the northwest region of the city, in neighborhoods home to many of the city’s residents of color.

However, without knowing the true rates of speeding violation—enforced or not—in different neighborhoods, we cannot say whether this gap stems from unequal enforcement or simply reflects differences in driving behavior by the city’s residents. For instance, in principle, there may be more speeding violations in Mesa’s northwest neighborhoods, in which case the observed racial and ethnic disparities in traffic stops could result from traffic enforcement that is proportional to traffic violations. Alternatively, however, actual speeding violations in majority minority neighborhoods may be comparable to, or lower than, speeding violations in predominately non-Hispanic White neighborhoods, in which case the disparities we see in speeding stops could reflect differential enforcement.

We disentangle these two possibilities by overlaying, in Fig. 1(B), a heatmap of actual speeding, as measured by our telematics data, where darker shades of orange indicate areas with larger numbers of speeding violations. The heatmap shows that speeding in Mesa is distributed across the city, and, importantly, is not restricted to the minority neighborhoods with high concentrations of police stops. In this case, it thus appears that police stops for speeding are driven in part by heavier enforcement in communities of color.

## Data and results

Our discussion of Mesa above demonstrates the importance of accounting for the true rates of speeding when assessing patterns of policing. We now augment this visual analysis with quantitative detail, and extend it to include 10 American cities: Aurora, CO; Chicago, IL; Houston, TX; Madison, WI; Mesa, AZ; Oklahoma City, OK; Plano, TX; San Antonio, TX; Tulsa, OK; and Wichita, KS. These cities were selected because of the availability of both telematics data, and high-resolution geocoded data on police stops for speeding, obtained from the Open Policing Project (21).

Our primary results are based on an aggregated telematics dataset from Cambridge Mobile Telematics (CMT). In total, we analyzed data from approximately 25,000,000 trips taken by 270,000 drivers in 2019 and early 2020—we limited the time range to circumvent the impacts of COVID-19 on driving behavior. For each police beat (or equivalent city-level jurisdiction, such as precinct), CMT computed the proportion of driving time that drivers spent driving 15 KPH (approximately 9 MPH) or more above the speed limit. Table 1 details summary population and traffic stop statistics for the 10 cities we analyzed.

**Table 1.** Summary statistics for the cities in our dataset, including the number of police beats, the mean and standard deviation of the residential population of each beat, the number of speeding and overall traffic stops in the city, and the proportion of traffic stops that were for speeding.

City	Beats	Avg. beat population		Speeding stops	Traffic stops	Prop. speeding (%)
Aurora	27	12,412	(SD: 5,537)	43,634	101,679	43
Chicago	268	10,024	(SD: 5,381)	55,303	1,738,393	3
Houston	98	20,971	(SD: 9,801)	214,226	725,442	30
Madison	104	2,184	(SD: 1,743)	29,742	107,072	28
Mesa	32	14,276	(SD: 7,428)	25,298	75,037	34
Oklahoma City	43	14,032	(SD: 7,551)	156,611	240,214	65
Plano	23	11,782	(SD: 5,640)	58,243	103,874	56
San Antonio	110	12,566	(SD: 6,180)	136,003	389,688	35
Tulsa	44	8,919	(SD: 3,506)	39,394	96,263	41
Wichita	36	9,782	(SD: 4,530)	83,088	196,881	42

One potential concern with using telematics data to estimate the frequency of speeding violations is that the sample of drivers for whom this information is available is not representative of the driving population. Suppose, for example, that telematics data are disproportionately available for drivers in certain neighborhoods. In that case, we might overestimate the frequency of speeding in those neighborhoods for the simple reason that we are measuring the behavior of a disproportionately large number of drivers in that location. To address this issue, we use the telematics data to instead measure the beat-specific rates of speeding, and then scale these estimates by the size of each beat’s residential population. This adjustment corrects for one of the most serious issues of nonrepresentativeness in our analysis. See “Materials and Methods” for further details and discussion on our approach to estimating total beat-specific driving time and time spent speeding from the telematics data.

**Fig. 2.**
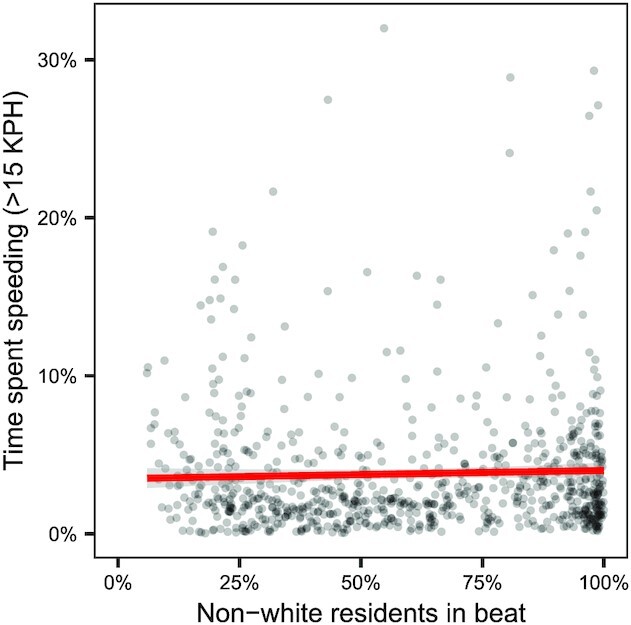
The proportion of driving time spent at least 15 KPH above the speed limit versus the proportion of non-White residents, where each point represents a police beat in the 10 cities we analyze. The flat line of best fit (in red) indicates that neighborhood composition and speeding behavior are largely unrelated (*r* = 0.04).

We begin our analysis by investigating the frequency of speeding in neighborhoods as a function of their demographic composition. (Throughout our analysis, we use demographic data from the US Census Bureau’s American Community Survey (22) for 2013 to 2018.) Specifically, for each beat in our analysis, we compute—based on the telematics data—the proportion of driving time spent at least 15 KPH over the posted speed limit. The results are shown in Fig. 2, where points correspond to beats across the 10 cities we consider. The nearly perfectly flat regression line indicates that speeding rates are largely unrelated to the demographic makeup of a neighborhood. As such, any observed disparities in stop rates are likely due to differences in enforcement rather than differences in violation rates.

To estimate the rate of enforcement as a function of beat demographics, after adjusting for speeding, we fit a negative binomial regression model. Specifically, we model the number of speeding stops in each beat in each year in our dataset as

(1) \begin{equation*} n_i \sim {\rm {N{\small EG}B{\small IN}}}(\mu _i e^{\beta _{{\rm R{\small ACE}},{\rm C{\small ITY}}[i]} r_i + \beta _{\rm D{\small RIVING}} d_i + \beta _{\rm Y{\small EAR}[i], \rm C{\small ITY}[i]}}, \phi ), \end{equation*}

where *i* indexes each observation (a beat in a particular year); *n_i_* is the number of recorded speeding stops; μ_*i*_ is the total amount of driving time in the beat (estimated with the telematics data); *r_i_* is the proportion of non-White residents in the beat and $\beta _{{\rm R{\small ACE}},\scriptsize{\rm C{\small ITY}}[i]}$ its corresponding coefficient, where each city has its own coefficient; *d_i_* is the proportion—normalized within each city to have mean 0 and variance 1—of time spent driving at least 15 KPH above the speed limit (estimated with the telematics data) and $\beta _{\scriptsize{\rm D{\small RIVING}}}$ its corresponding coefficient; $\beta _{\scriptsize{\rm Y{\small EAR}}[i], \scriptsize{\rm C{\small ITY}}[i]}$ is a fixed effect to allow for policing practices that vary between years and cities; and ϕ is the dispersion parameter, which controls the variance of the distribution. (Throughout this paper, we use ‘White’ to refer to non-Hispanic White individuals and ‘non-White’ to refer to American Indian or Alaska Native, Asian, Black or African American, Hispanic or Latino, or Native Hawaiian or other Pacific Islander individuals according to the US Census (23).)

The main terms of interest in our model are the values of $\hat{\beta }_{\rm R{\small ACE}}$ for each city, which capture the estimated rate of speeding stops in a beat as a function of its proportion of non-White residents after adjusting for driving time and speeding behavior. Positive values of $\hat{\beta }_{\rm R{\small ACE}}$ indicate that beats with higher proportions of non-White residents have disproportionately high numbers of speeding stops relative to driving behavior.

**Fig. 3.**
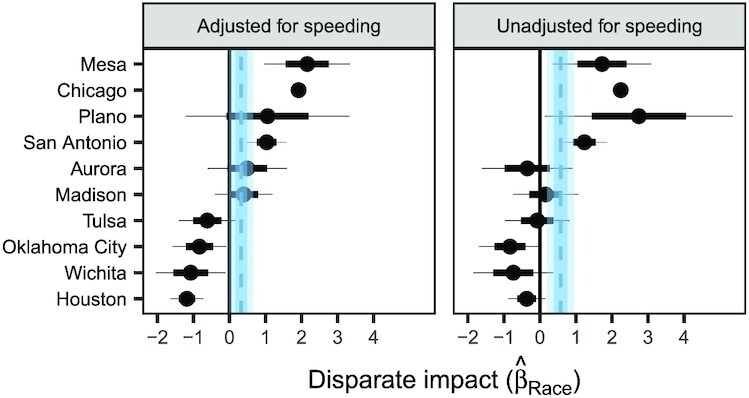
The value of $\hat{\beta }_{\rm R{\small ACE}}$ in our regression for each city, adjusting for the true prevalence of speeding (left) versus raw, unadjusted results (right). In both the adjusted and unadjusted regressions, we find significant heterogeneity across cities, suggesting that the nature and degree of policing practices varies considerably between cities. The mean of the unadjusted city-level coefficients is 0.57 (SE: 0.18, *P* = 0.002), meaning that speeding enforcement tends to be higher in areas with greater proportions of non-White residents. After adjusting for speeding, our results are qualitatively similar, with an average city-level coefficient of 0.34 (SE: 0.16, *P* = 0.044), meaning that disparities in speeding enforcement remain even after adjusting for driving behavior.

The city-level values of $\hat{\beta }_{\rm R{\small ACE}}$ are shown in Fig. 3 (left panel). We find a wide range of coefficients between the cities, from 2.16 (SE: 0.60, *P* < 0.001) in Mesa to −1.18 (SE: 0.23, *P* < 0.001) in Houston. These results suggest that in some cities, such as Mesa, speeding enforcement is higher in neighborhoods with larger non-White populations, whereas in other cities, such as Houston, enforcement is higher in neighborhoods with larger White populations. Taking the unweighted average of the city-level coefficients across the 10 cities we study, we estimate an overall $\hat{\beta }_{\rm R{\small ACE}}$ equal to 0.34 (SE: 0.17, *P* = 0.044). (We computed the standard error using the method of the sim function in the armR package (24), which accounts for the covariance structure of the city-level coefficients. See Table S2 for the complete regression table.) Thus, on average across the cities we examine, and after adjusting for driving behavior, a beat for which 75% of its residents are non-White experiences approximately 20% more speeding stops than a beat in which 75% of its residents are white. Finally, we note that $\hat{\beta }_{\rm D{\small RIVING}} = 0.54$ (SE: 0.018, *P* < 0.001), suggesting that, on average across the cities we study, enforcement is indeed higher in areas with higher amounts of speeding.

As described in the Supplementary Information, these results are robust to differences in the choice of speeding threshold (i.e. to differences in how one might operationalize speeding behavior, as shown in Figure S1). We likewise find qualitatively similar results when we repeat our analysis using quasipoisson regression, instead of negative binomial regression (Figure S2). Further, repeating our analysis with an alternate source of telematics data from TomTom, we find a similar degree of heterogeneity between cities, but somewhat higher values of $\hat{\beta }_{\rm R{\small ACE}}$ across cities (see Figures S13–S18).

As a point of comparison, the right panel of Fig. 3 shows the results of a regression that does not adjust for speeding. The estimated race coefficients are broadly similar to those from the model above that does adjust for speeding, a pattern we would expect given that speeding is largely unrelated to beat demographics, as shown in Fig. 2. In other words, raw disparities in enforcement practices largely persist after adjusting for driving behavior (cf. Table S1).

**Fig. 4.**
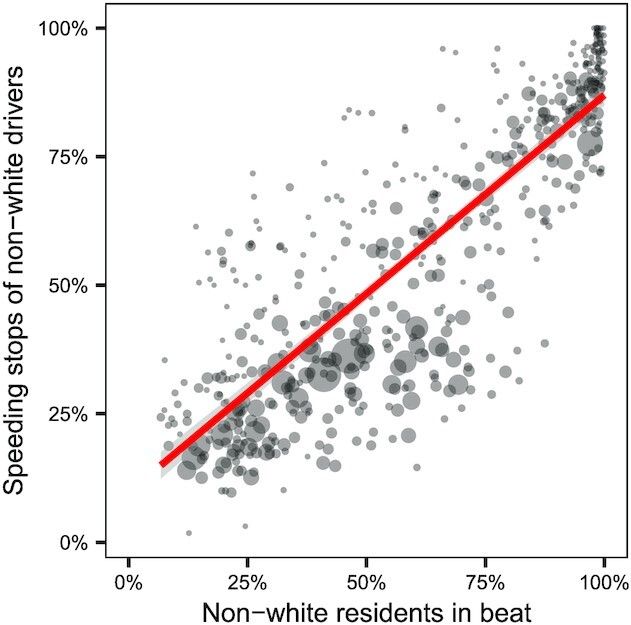
For each beat in our dataset, the proportion of non-White residents in the beat versus the proportion of drivers stopped for speeding who were non-White, along with a line of best fit in red. The size of the dots are proportional to the number of drivers stopped for speeding in the beat. We find that the residential demographics of a beat are largely reflected in the demographics of drivers stopped for speeding (*r* = 0.83).

Our analysis thus far has focused on connections between beat-level driving behavior and beat-level police enforcement, due to the deidentified and aggregated nature of our telematics data, which do not contain driver demographics. However, as we show in Fig. 4, the racial composition of drivers stopped in a beat is strongly correlated with the racial composition of the beat’s residential population (*r* = 0.83). These results suggest that when enforcement is concentrated in racially nonrepresentative beats, the enforcement gap translates to racial disparities among stopped drivers.

**Fig. 5.**
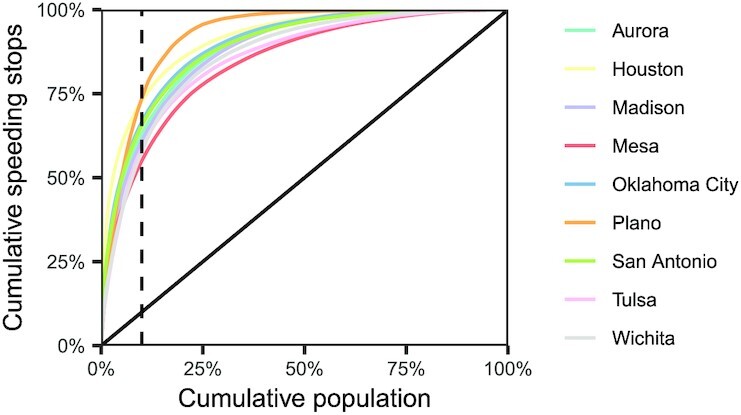
For each city in our analysis, the cumulative proportion of speeding stops as a function of the cumulative population of the US Census block groups they occur in. We find that speeding stops are concentrated in block groups containing a relatively small proportion of the population. Concretely, block groups containing just 10% of the residential population, indicated by the dashed vertical line, contain between 56% and 76% of the total speeding stops across the cities we study.

Our results above indicate that while many cities exhibit racial disparities in speeding enforcement, those disparities do not consistently impact a single demographic group. This pattern appears to stem in part from the concentration of speeding enforcement in relatively small areas of the cities, and, in many cases, areas that are demographically nonrepresentative of the cities overall. For each of the 10 cities we analyzed, Fig. 5 plots the cumulative number of speeding stops in the top *k* locations, defined in terms of US Census block groups. Across cities, locations accounting for just 10% of the residential population contain between 56% and 76% of speeding stops. This concentration implies that even small deviations in where police departments choose to heavily enforce speeding can lead to substantial demographic disparities, an effect that is exacerbated by geographic segregation within cities. Figures S3–S11 visually depict the concentration of speeding stops, and their connection to population demographics, in each of the cities we analyzed.

Our analysis has focused primarily on the enforcement of speeding violations, finding that, in some cites, speeding is more heavily enforced in predominately non-White neighborhoods, while, in others, speeding is more heavily enforced in predominately white neighborhoods. Speeding, however, is just one of many stated reasons for a traffic stop—other common reasons include equipment violations, such as broken taillights. Indeed, we find that the proportion of traffic stops for speeding varies widely between cities in our analysis, ranging from 3% in Chicago to 65% in Oklahoma City. (Table 1 contains statistics for all the cities we consider.)

We conclude by quantifying the aggregate disparities stemming from traffic stops as a whole, not simply stops for speeding. By necessity, this exercise differs from our analysis above, in that we are not able to estimate the underlying prevalence of nonspeeding violations. We, thus cannot disentangle whether any observed disparities are due to differences in enforcement as opposed to differences in rates of infraction. Specifically, we model the number of traffic stops as

(2) \begin{equation*} v_i \sim \rm N{\small EGBIN}(\mu _i e^{ \beta _{\rm R{\small ACE},\rm C{\small ITY}[i]} r_i + \beta_{\rm Y{\small EAR}[i],\rm C{\small ITY}[i]} }, \phi ), \end{equation*}

where *v_i_* is the total number of traffic stops in the beat associated with observation *i*, and the remaining terms are defined as in (1).

**Fig. 6.**
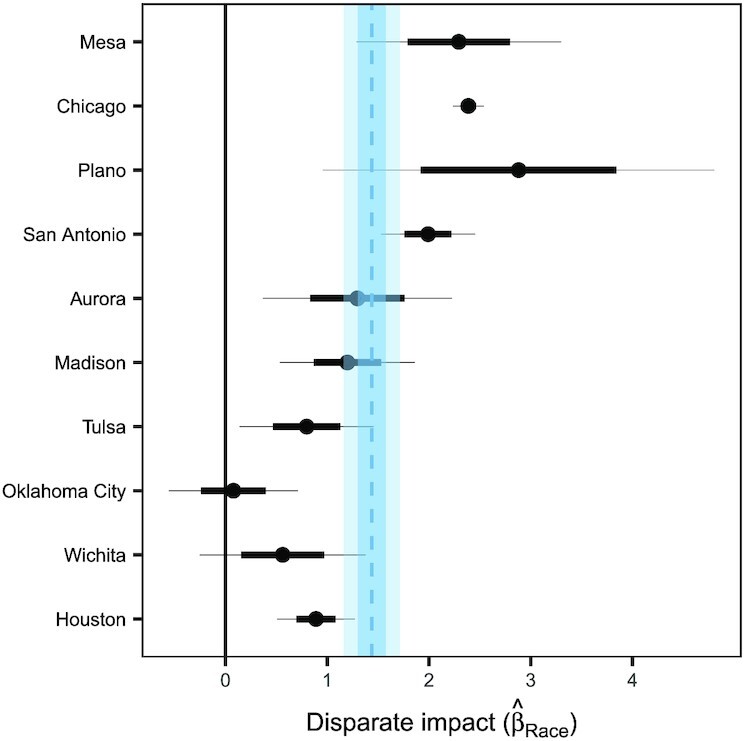
The city-level values of $\hat{\beta }_{\rm R{\small ACE}}$ in our regression for all traffic violations. We find uniformly positive coefficients with average 1.44 (SE: 0.14, *P* < 0.001), meaning that in every city we analyze, there were more recorded traffic violations in beats with higher proportions of non-White residents when not adjusting for true prevalence of violations.

The city-level values of $\hat{\beta }_{\rm R{\small ACE}}$ for the above regression are shown in Fig. 6. In contrast to our analysis of speeding stops, all 10 cities we examine yield positive coefficients, with statistical significance in eight of the cases. Averaged across cities, we estimate an overall $\hat{\beta }_{\rm R{\small ACE}}$ of 1.33 (SE: 0.15, *P* < .001). (As above, we compute the standard error in a manner that accounts for the covariance structure of the city-level coefficients.) While this particular analysis has not accounted for the underlying infraction rates, we believe it is an important data point for understanding the full impacts of policing, particularly on communities of color.

## Discussion

Combining aggregated telematics data on tens of millions of trips with records on nearly 800,000 police stops for speeding in 10 American cities, we compare the enforcement of speeding violations with the true, underlying violation rates. Averaged across the cities we analyze, and after adjusting for differences in speeding behavior, we find that neighborhoods with higher proportions of non-White residents have greater numbers of speeding stops. We also, however, find substantial variation across cities, and, in several cities, speeding is more heavily enforced in predominantly white neighborhoods. We trace this heterogeneity back in part to the heavy concentration of police stops in a relatively small number of neighborhoods. Given the levels of residential segregation common in many American cities, decisions in where to patrol can, thus lead to substantial disparities in who is stopped.

One limitation with our methodological approach is that the sample of drivers for whom we have telematics data is not necessarily representative of the driving population as a whole (see Figure S12). We address this concern in part by reweighting our sample (as described in the “Methods and Materials”) to better reflect the residential population. It is, however, still possible that CMT users are, on average, safer drivers overall. Our analysis only requires estimates of the relative violation rates across beats, but even this quantity might not be accurately captured by our data. To assess the sensitivity of our results to this possibility, we repeat our analysis on an alternative source of telematics data, from TomTom—described in the “Methods and Materials”—and obtain broadly similar results. Although it is possible that the TomTom data also suffer from some bias, the concordance of results across datasets provides added evidence for the robustness of our findings.

Our results suggest that, in many cases, speeding enforcement leads to disparate impacts that are not accounted for by underlying driving behavior. That is, while levels of speeding in a location are largely unrelated to the neighborhood’s demographic composition, enforcement of speeding is often concentrated in demographically nonrepresentative areas. It bears emphasizing, though, that such disparate impacts do not imply discriminatory intent. The patterns we see may—at least in part—be the unintended consequence of a complex series of policy choices.

Regardless of motive, the impacts of differential enforcement can often place undue burdens on communities, particularly communities of color (25,26). Aside from the immediate financial costs of speeding tickets, late or missed payments—common among economically disadvantaged subgroups—can lead to substantial fines and fees, revocation of driving privileges, and, in some instances, incarceration (27,28). Traffic stops can also escalate to more serious encounters, with about 1 in 15 stops involving use of force (1). Further, traffic stops can prompt disproportionate enforcement of other laws, with increased scrutiny of stopped drivers potentially uncovering evidence of offenses—such as expired driver’s licenses, minor drug possession, and missed court appointments—that would have otherwise gone unnoticed (29). Frequent police contact can also deteriorate trust in institutions and exacerbate “system avoidance,” with individuals less willing to seek out government services, contributing to social stratification (30). In addition to these potential harms of over-policing traffic violations, under-policing can also create equity issues by heightening risks for both drivers and pedestrians in neighborhoods with lax enforcement (31).

Our results point to the importance of reconsidering current traffic enforcement policies. In some cases, jurisdictions may be able to maintain traffic safety while simultaneously avoiding some of the adverse consequences of policing by adopting less punitive measures—for example, by deterring unsafe driving through speed bumps and roundabouts rather than citations. We further note that a policy or practice of concentrating policing in small regions of a city makes it more likely that the affected drivers are a demographically nonrepresentative subset of the city’s residents. Although such concentration may have certain benefits of efficiency, it is equally important to design enforcement strategies with an eye toward ensuring equity. Some scholars have even argued for more explicitly randomized policing strategies to combat the inherent inequities of targeted action (32).

Finally, it is important to recognize that a city’s physical infrastructure can itself exacerbate disparities attributable to enforcement policies. In particular, there is a long history of building highways, which displace and segregate Black communities in the United States (33,34); when such discriminatory policies increase distance between those communities and “society’s opportunity” (35,36), they can increase the time Black drivers spend on the road, and thus their potential for being stopped for speeding. Looking forward, we hope our methodological approach and substantive findings help researchers, policymakers, and advocates better evaluate policing practices, and, in turn, design more equitable policies.

## Materials and methods

### Telematics data

We used deidentified and aggregated telematics data from CMT. CMT stores data at the level of “trips,” where each trip records a single drive by a single CMT user, and consists of a series of *waypoints* (pairs of latitudes and longitudes) with timestamps recording when drivers passed through given locations. Using these waypoints, one can estimate the speed at which a driver was travelling at each moment. For each beat, CMT aggregated the data to compute the total amount of time drivers spent driving in the beat, and the total amount of time drivers spent driving at various thresholds above the posted speed limit; the ratio of these terms yields an estimate of the proportion of time drivers spent speeding in the beat—the normalized version of this proportion is what we call *d_i_* in our primary regression. We excluded driving occurring on interstate highways and other freeways and expressways—as defined by the Federal Highway Administration (37)—to focus our analysis on local drivers, whose demographic composition more closely resembles the demographic composition of residents of their neighborhood. For the same reason, we also excluded beats which consisted of airports, lakes, and other nonresidential areas.

Key to our analytic approach is estimating the relative amount of time spent driving in each beat—μ_*i*_ in our regressions. However, the recorded amount of time CMT users spent in each beat, by itself, can yield biased estimates, especially if the population of CMT users is not a demographically representative sample of the residential population. To adjust for this possibility, CMT reweighted the driving data so that the distribution of drivers’ home beats in the weighted sample mirror the distribution of home beats in the residential population.

Specifically, for each driver *k*, CMT first imputed their home beat *h_k_* as the beat in which the driver’s car was parked for the longest period of time. In order to ensure that drivers’ home beats could be imputed reasonably accurately, the analysis was restricted to drivers for whom the data included at least 10 trips, comprising 52% of the total number of drivers present in the data, who were collectively responsible for 98% of the total trips present in the data. Then, for each pair of beats (*i, j*), CMT computed the average amount of time *a_ij_* that drivers with home beat *i* spent driving in beat *j* per day. Finally, the adjusted driving time, μ_*j*_, in each beat was computed as follows: $\mu _j = \sum _{i=1}^N a_{ij} \cdot p_i$, where *p_i_* is the residential population of beat *i*.

### Police stop data

We use police stop data from the Open Policing Project (21), a repository of records from 35 municipal police departments across the United States. We filtered to cities where: (1) telematics data were available; (2) the city contained at least five beats; (3) the beat in which a stop occurred can be consistently inferred, either because it is explicitly recorded, or because it can be imputed from recorded latitudes and longitudes; (4) the reason for the stop (e.g. “speeding”) was recorded; and (5) at least 1,000 speeding stops were present in the data. After applying these filters, we were left with the 10 cities comprising our primary analysis. Mirroring our restriction of the telematics data, we excluded stops occurring on highways.

### Robustness checks

We ran a series of robustness checks, described below, to gauge the sensitivity of our results to varying datasets and analytic assumptions. In all cases, we obtained results that were broadly similar to our main results reported above.

First, we examined the robustness of our results to the particular source of telematics data. To do so, we used publicly available data from the TomTom Traffic Stats API. We obtained speed ventiles and the number of vehicles, which passed through each road segment in our 10 cities for the month of April 2019. Due to the aggregated nature of the TomTom data, our estimates of time spent driving and time spent speeding in each beat differed from that in our primary analysis. In particular, we could not infer driver home beats, and consequently could not adjust for potential nonrepresentativeness among the sample of TomTom drivers. We computed an alternative measure of time spent speeding in each beat by taking a weighted average over each road segment in the beat of the proportion of time spent going 15 KPH above the speed limit, weighted by the length of the road segment. We likewise computed an alternative measure of total drive time, $\tilde{\mu }_i = \sum _j n_j l_j$, where *j* indexes over all road segments within beat *i, n_j_* is the number of vehicles, which passed through road segment *j*, and *l_j_* is the length of the *j*th road segment. The city-level estimates of $\beta _{\rm R{\small ACE}}$ using the TomTom data are displayed in Figure S14. The estimated coefficients are qualitatively similar to those found with the CMT data—including the heterogeneity across cities—but the overall average is larger with the TomTom data than with the CMT data, suggesting larger average speeding enforcement disparities in communities of color.

Second, we assess the sensitivity of our results to the precise definition of “speeding.” Driving at any speed above the speed limit is a ticketable offense; however, police may, for instance, have a policy of only pulling over motorists who speed excessively. For this reason, we define “speeding”, in our main analysis, as travelling at least 15 KPH (approximately 9 MPH) over the posted speed limit. Figure S1 shows that our primary results are consistent over a wide range of thresholds. Similarly, it is possible that police officers target speeders not based on the absolute degree to which they are speeding, but rather how much they are speeding relative to the speed limit. For instance, a motorist driving at 22 MPH on a 15-MPH road may be pulled over, while a motorist driving 62 MPH on a 55-MPH road may not be. Consequently, we also consider relative speeding thresholds, where we consider a driver to be speeding if they exceed a certain percentage of the posted speed limit (e.g. driving more than 10% faster than the limit). Figure S1 again shows that our main results hold when we define speeding in terms of relative rather than absolute thresholds.

Finally, we re-ran our regression analysis with quasipoisson models, rather than the negative binomial models presented in the main text. The results are shown in Figure S2. We find that our primary results are qualitatively similar, except that the estimate of $\beta _{\rm R{\small ACE}}$ in Houston, 0.036 (SE: 0.181, *P* = 0.843), is no longer statistically significant.

## Supplementary Material

pgac144_Supplemental_Files

## Data Availability

Our analysis code, police stop and citation data, and the aggregated TomTom telematics data are publicly available at https://github.com/stanford-policylab/telematics. Due to privacy concerns, we are unable to release telematics data from Cambridge Mobile Telematics, but we note that the TomTom telematics data are sufficient to replicate our main results.
