# Supplementary material for: Measuring racial and ethnic disparities in traffic enforcement with large-scale telematics data
Source: PNAS Nexus. 2022 Jul 30;1(4):pgac144. doi: 10.1093/pnasnexus/pgac144 (PMC9802422; doi:10.1093/pnasnexus/pgac144)
Supplement: pgac144_Supplemental_Files [file pgac144_supplemental_files.zip › PNASNEXUS-PNASNEXUS-2021-00197-s02.pdf]

Supplementary Information for

Measuring Racial Disparities in Traffic Enforcement with Large-Scale Telematics Data

William Cai, Johann Gaebler, Justin Kaashoek, Lisa Pinals, Samuel Madden, Sharad Goel

William Cai (willcai@stanford.edu)
Sharad Goel (sgoel@hks.harvard.edu)

This PDF file includes:

Figs. S1 to S18
Tables S1 to S2

Table S1. To corroborate our main analysis, we compare the ratio of stopped drivers per capita by race with our regression coefficients. We note that within a single city, the per capita ratio of stopped drivers is not expected to be unity due to white drivers on average driving more (Kim, W., V. Anorve, and B. C. Tefft. "American Driving Survey, 2014–2017." (2019)). Furthermore, neither Oklahoma City nor Houston coded Hispanic drivers in their stop data, and so we excluded them here. We find that proportionally more stopped speeders tend to be white in cities with negative values for β_{RACE} and proportionally more tend to be non-white in cities with positive β_{RACE} coefficients. This is consistent with the interpretation of greater values of β_{RACE} as concentration of speeding enforcement on speeding by non-white drivers.

City	Black/white ratio	Hispanic/white ratio	Coefficient (SE)
Mesa	1.54	0.47	2.16 (0.60)
Chicago	2.44	0.79	1.92 (0.093)
Plano	1.55	0.50	1.06 (1.14)
San Antonio	1.12	0.92	1.03 (0.27)
Aurora	0.99	0.06	0.49 (0.55)
Madison	2.28	1.00	0.40 (0.40)
Tulsa	0.72	0.04	-0.62 (0.39)
Oklahoma City	0.83		-0.83 (0.37)
Wichita	0.96	0.58	-1.08 (0.48)
Houston	0.59		-1.18 (0.23)

Table S2. Regression table for the main analysis. The variable "Speeding over 15 KPH" is the normalized percent of time drivers spent driving in a beat at least 15 KPH over the speed limit, and "% Non-White" is the percentage of residents of a beat who are not non-Hispanic white. City names represent city fixed effects, years represent year fixed effects, and interactions are indicated by "×."

Term	Estimate	Standard Error	P-value
Speeding Over 15 KPH	0.542	0.018	0.000
Aurora	0.420	0.371	0.258
Chicago	-4.586	0.098	0.000
Houston	1.931	0.205	0.000
Madison	0.826	0.146	0.000
Mesa	-0.306	0.320	0.338
Oklahoma	0.821	0.250	0.001
Plano	2.451	0.542	0.000
San Antonio	-1.060	0.234	0.000
Tulsa	0.578	0.244	0.018
Wichita	1.361	0.268	0.000
Aurora × % Non-White	0.492	0.549	0.370
Chicago × % Non-White	1.918	0.093	0.000
Houston × % Non-White	-1.183	0.228	0.000
Madison × % Non-White	0.395	0.399	0.321
Mesa × % Non-White	2.159	0.595	0.000
Oklahoma × % Non-White	-0.833	0.374	0.026
Plano × % Non-White	1.058	1.138	0.353
San Antonio × % Non-White	1.032	0.274	0.000
Tulsa × % Non-White	-0.617	0.392	0.115
Wichita × % Non-White	-1.076	0.483	0.026
Aurora × 2014	-0.105	0.296	0.722
Chicago × 2014	0.418	0.098	0.000
Houston × 2014	0.227	0.155	0.144
Madison × 2014	-0.200	0.156	0.200
Mesa × 2014	-0.127	0.272	0.641
Oklahoma × 2014	0.007	0.234	0.976
Plano × 2014	0.025	0.320	0.937
San Antonio × 2014	-0.146	0.146	0.320
Tulsa × 2014	-0.171	0.232	0.461
Wichita × 2014	0.075	0.256	0.771
Aurora × 2015	-0.231	0.296	0.435
Chicago × 2015	-0.022	0.098	0.819
Houston × 2015	0.072	0.155	0.644
Madison × 2015	0.072	0.156	0.646
Mesa × 2015	0.253	0.272	0.352
Oklahoma × 2015	-0.162	0.234	0.488
San Antonio × 2015	-0.336	0.147	0.022
Tulsa × 2015	-0.381	0.232	0.100
Wichita × 2015	0.100	0.256	0.695
Aurora × 2016	-0.362	0.296	0.221
Chicago × 2016	0.293	0.098	0.003
Houston × 2016	-0.045	0.155	0.770
Madison × 2016	-0.202	0.156	0.196
Oklahoma × 2016	-0.129	0.234	0.583
San Antonio × 2016	-0.214	0.146	0.143
Tulsa × 2016	-0.042	0.232	0.857
Wichita × 2016	0.023	0.256	0.929
Chicago × 2017	0.649	0.097	0.000
Oklahoma × 2017	-0.424	0.234	0.070
San Antonio × 2017	-0.387	0.147	0.008
Chicago × 2018	0.544	0.097	0.000
Chicago × 2019	0.537	0.097	0.000

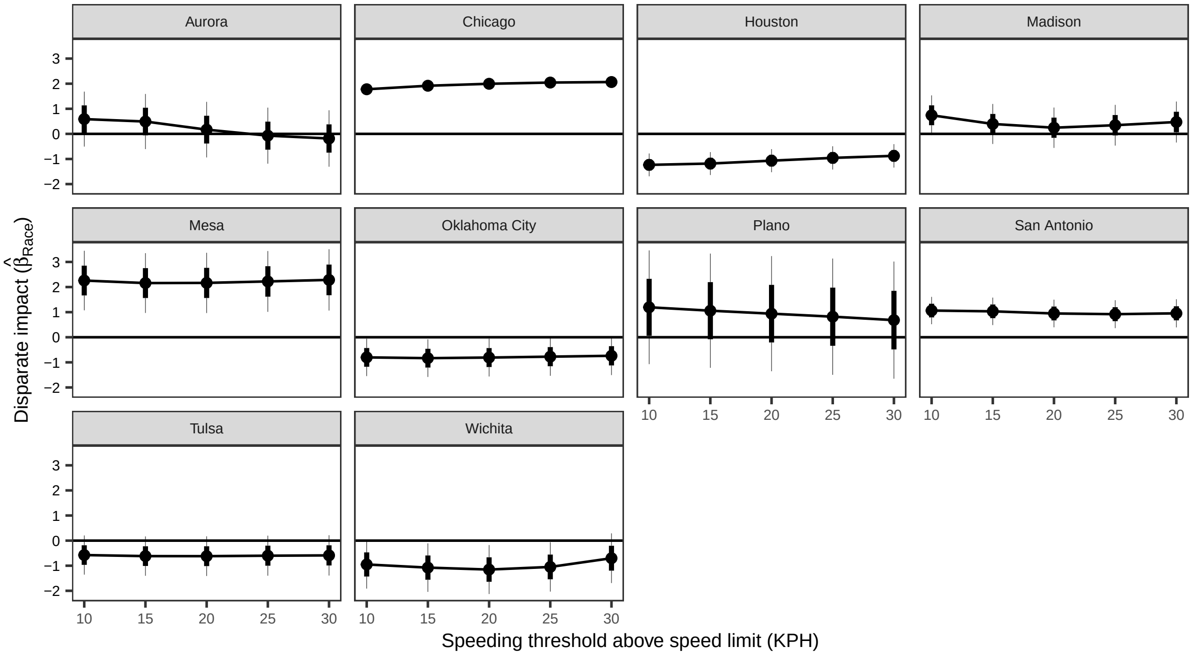

(a) Absolute speeding thresholds

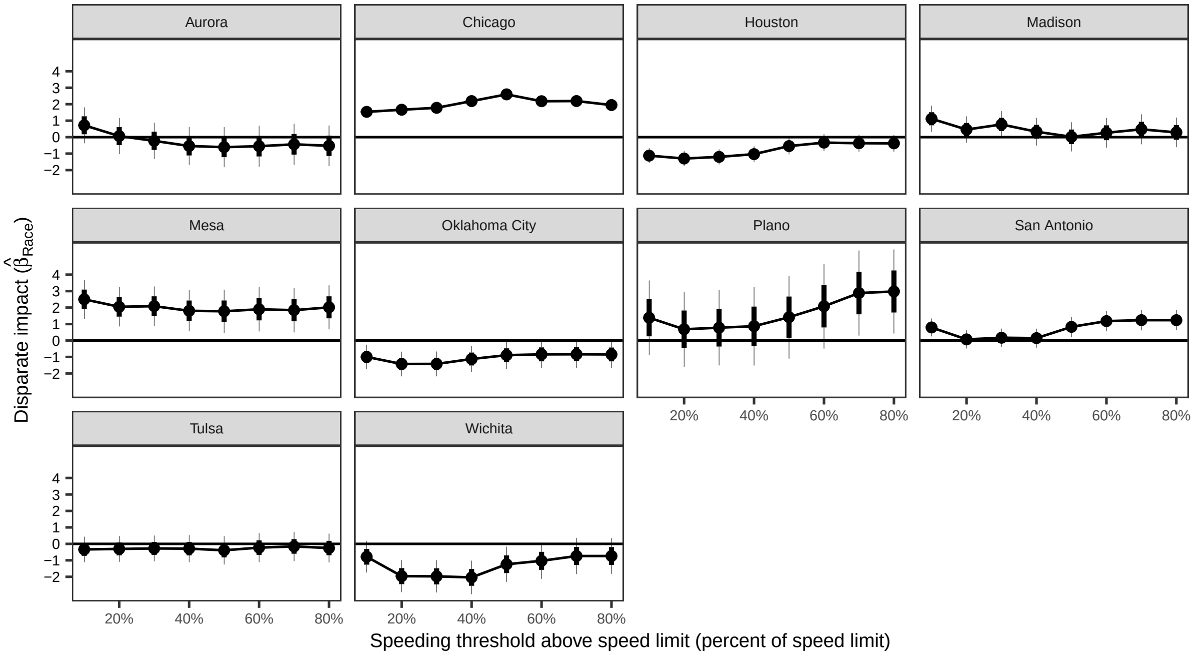

(b) Relative speeding thresholds

Fig. S1. Robustness of our main analysis to the definition of speeding. We show, for each city, the value of β_{RACE} as we vary the threshold above the speed limit we consider speeding when we compute d_i, on an absolute (S1a) and relative (S1b) scale. We find that our estimates of the coefficients are largely stable as we shift the threshold.

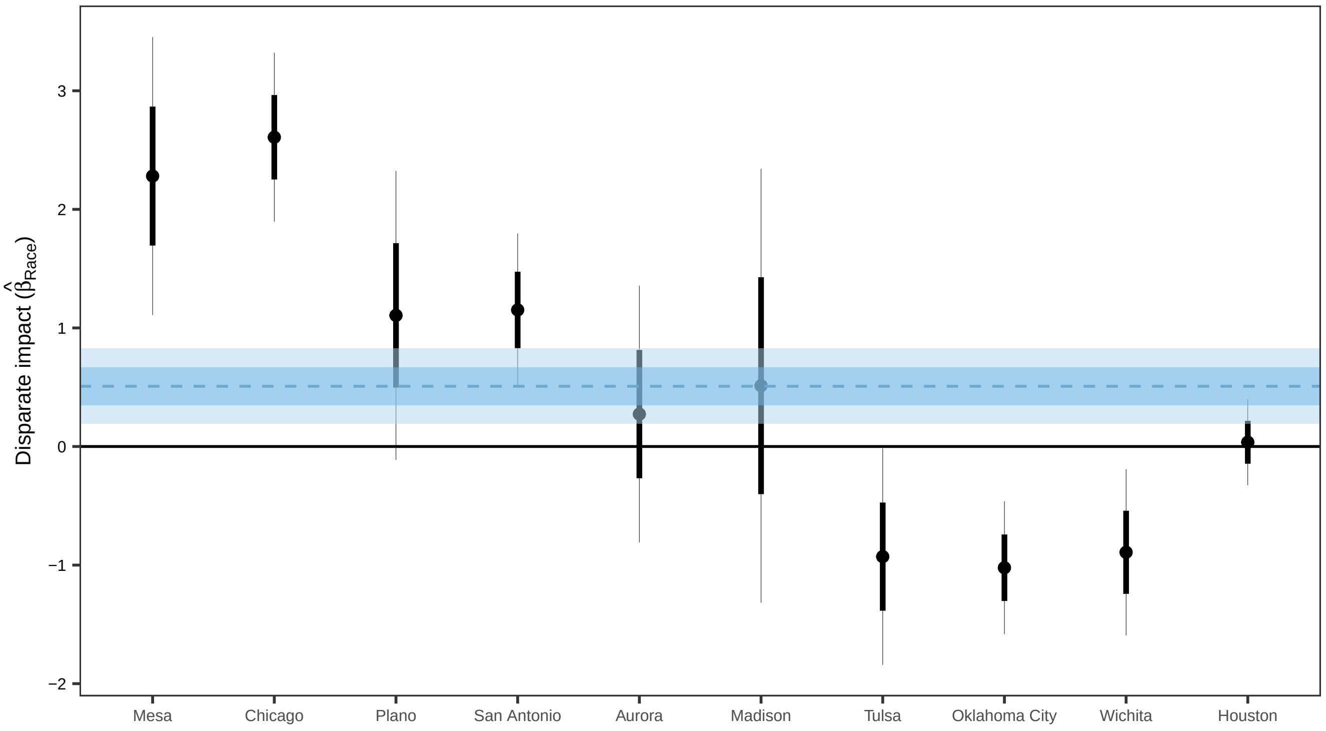

Fig. S2. Estimates of β_{RACE} in our speeding violation analysis using quasipoisson instead of negative binomial regression. We find that our results are largely unchanged, except that Houston moves from having a negative coefficient in the main model to 0.036 (SE: 0.181, $p = 0.843$).

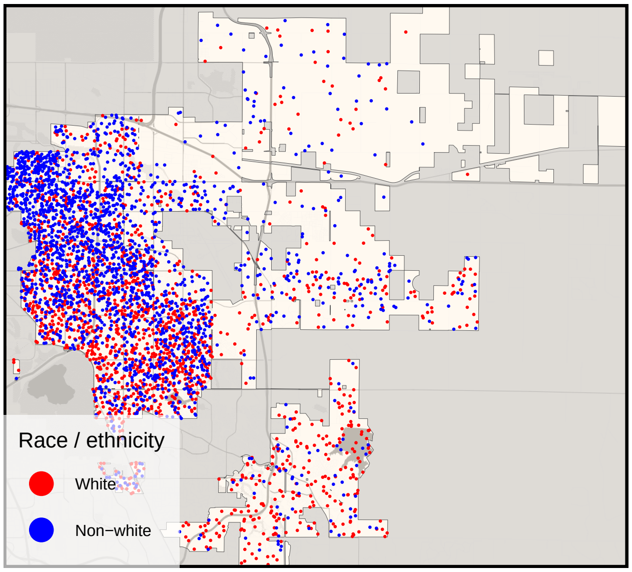

(a) Demographics.

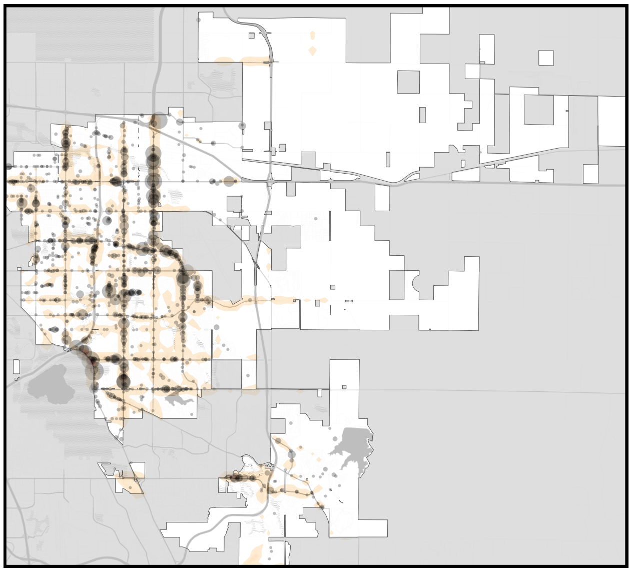

(b) Speeding violations and stops.

Fig. S3. A comparison of Aurora, CO's demographic composition, the location of speeding events, as measured by telematics data, and the location of speeding stops. (S3a): The demographics of residents, where red points represent non-Hispanic white residents and blue points represent non-white residents. (S3b): The density of speeding stops recorded by police officers, indicated by gray dots, where the area of the points is proportional to the number of stops at a single location; stops for speeding are overlayed with a heatmap of speeding events, as estimated from telematics data.

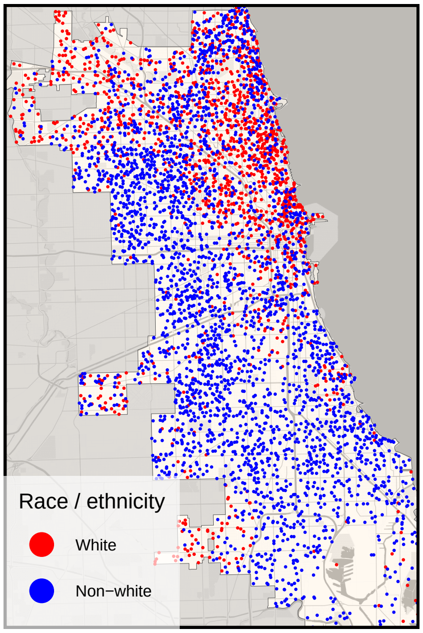

Fig. S4. The demographics of residents of Chicago, IL, where red points represent non-Hispanic white residents, green points Black residents, blue points Hispanic residents, purple points Asian and Pacific Islander residents, and orange points all other residents. (Traffic stops in Chicago indicate beat but were not geocoded.)

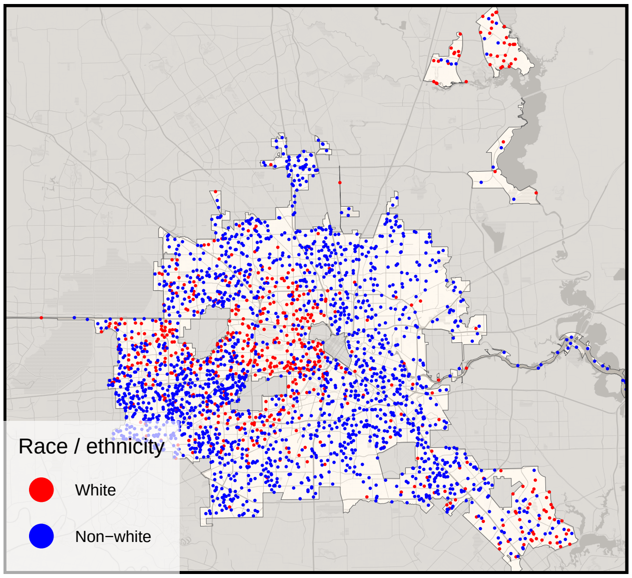

(a) Demographics.

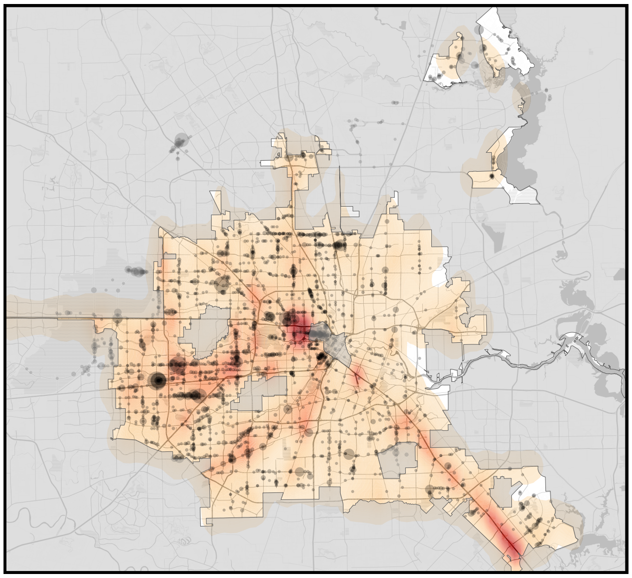

(b) Speeding violations and stops.

Fig. S5. A comparison of Houston, TX's demographic composition, the location of speeding events, as measured by telematics data, and the location of speeding stops. (S5a): The demographics of residents, where red points represent non-Hispanic white residents and blue points represent non-white residents. (S5b): The density of speeding stops recorded by police officers, indicated by gray dots, where the area of the points is proportional to the number of stops at a single location; stops for speeding are overlayed with a heatmap of speeding events, as estimated from telematics data.

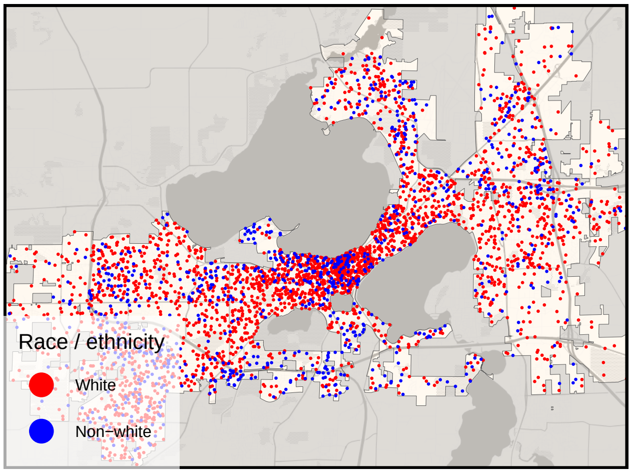

(a) Demographics.

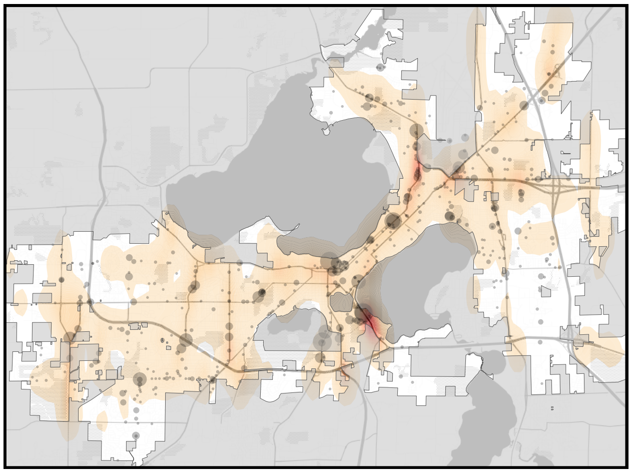

(b) Speeding violations and stops.

Fig. S6. A comparison of Madison, WI's demographic composition, the location of speeding events, as measured by telematics data, and the location of speeding stops. (S6a): The demographics of residents, where red points represent non-Hispanic white residents and blue points represent non-white residents. (S6b): The density of speeding stops recorded by police officers, indicated by gray dots, where the area of the points is proportional to the number of stops at a single location; stops for speeding are overlayed with a heatmap of speeding events, as estimated from telematics data.

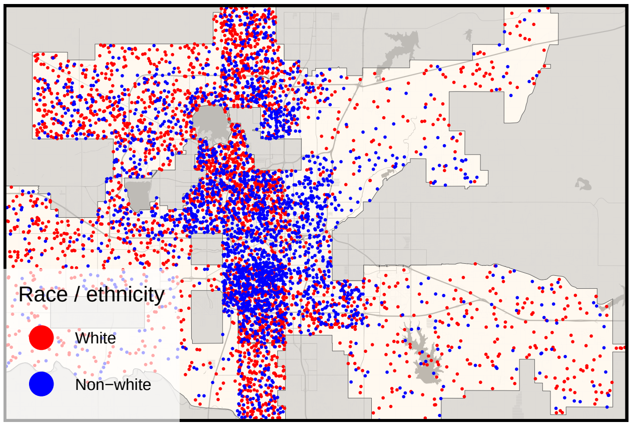

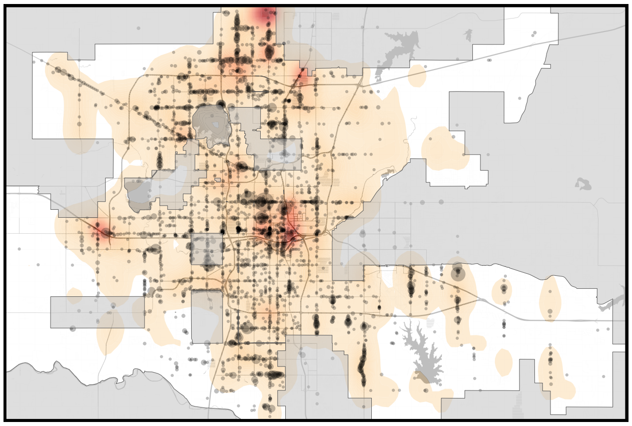

(a) Demographics.

(b) Speeding violations and stops.

Fig. S7. A comparison of Oklahoma City, OK's demographic composition, the location of speeding events, as measured by telematics data, and the location of speeding stops. (S7a): The demographics of residents, where red points represent non-Hispanic white residents and blue points represent non-white residents. (S7b): The density of speeding stops recorded by police officers, indicated by gray dots, where the area of the points is proportional to the number of stops at a single location; stops for speeding are overlayed with a heatmap of speeding events, as estimated from telematics data.

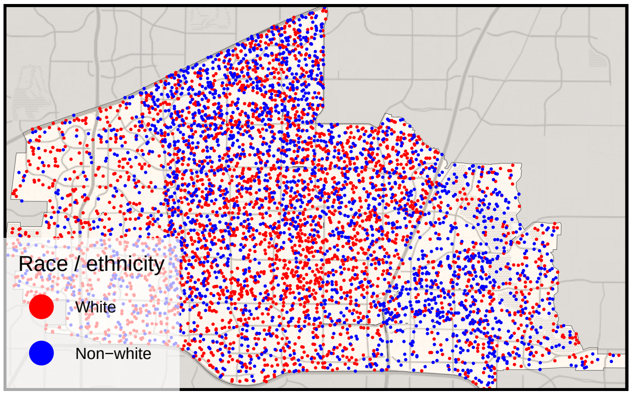

(a) Demographics.

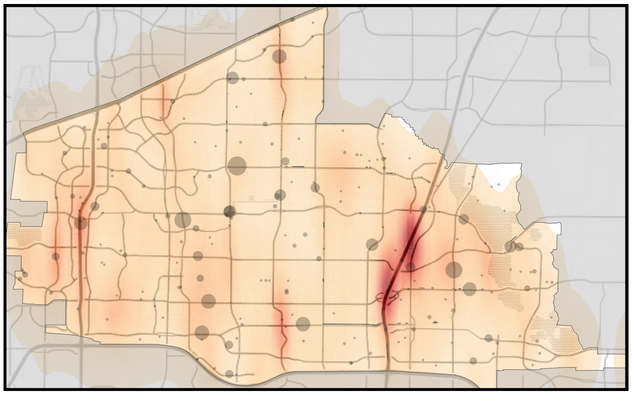

(b) Speeding violations and stops.

Fig. S8. A comparison of Plano, TX's demographic composition, the location of speeding events, as measured by telematics data, and the location of speeding stops. (S8a): The demographics of residents, where red points represent non-Hispanic white residents and blue points represent non-white residents. (S8b): The density of speeding stops recorded by police officers, indicated by gray dots, where the area of the points is proportional to the number of stops at a single location; stops for speeding are overlayed with a heatmap of speeding events, as estimated from telematics data.

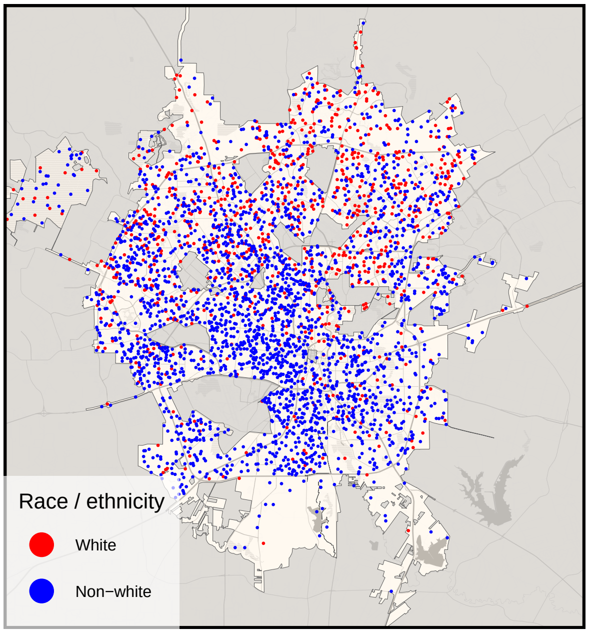

(a) Demographics.

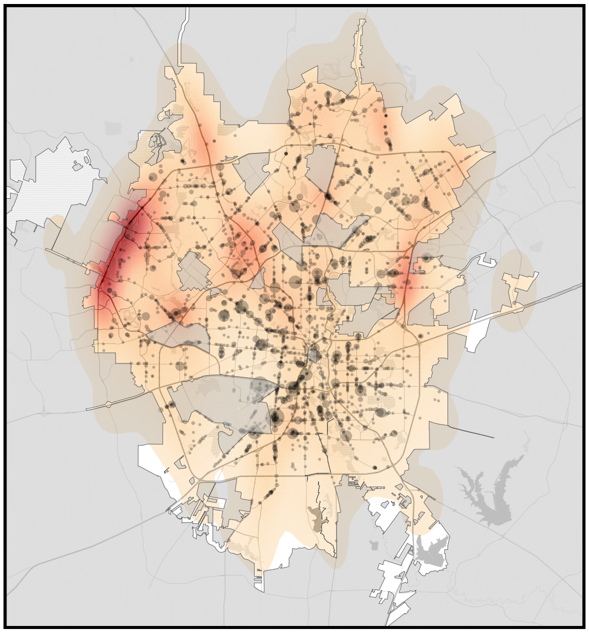

(b) Speeding violations and stops.

Fig. S9. A comparison of San Antonio, TX's demographic composition, the location of speeding events, as measured by telematics data, and the location of speeding stops. (S9a): The demographics of residents, where red points represent non-Hispanic white residents and blue points represent non-white residents. (S9b): The density of speeding stops recorded by police officers, indicated by gray dots, where the area of the points is proportional to the number of stops at a single location; stops for speeding are overlayed with a heatmap of speeding events, as estimated from telematics data.

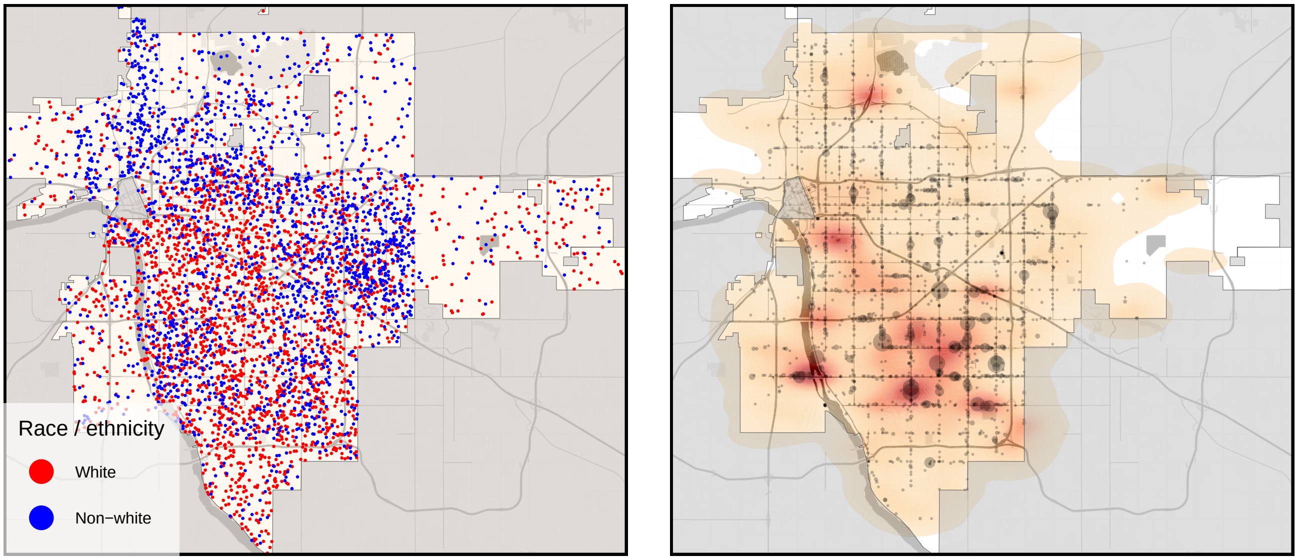

(a) Demographics.

(b) Speeding violations and stops.

Fig. S10. A comparison of Tulsa, OK's demographic composition, the location of speeding events, as measured by telematics data, and the location of speeding stops. (S10a): The demographics of residents, where red points represent non-Hispanic white residents and blue points represent non-white residents. (S10b): The density of speeding stops recorded by police officers, indicated by gray dots, where the area of the points is proportional to the number of stops at a single location; stops for speeding are overlayed with a heatmap of speeding events, as estimated from telematics data.

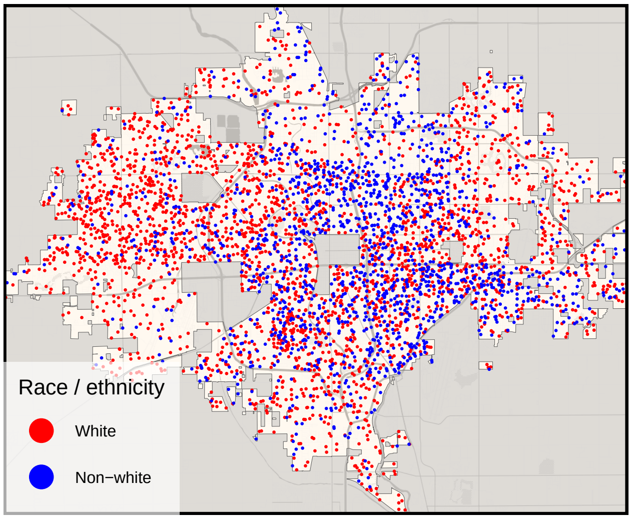

(a) Demographics.

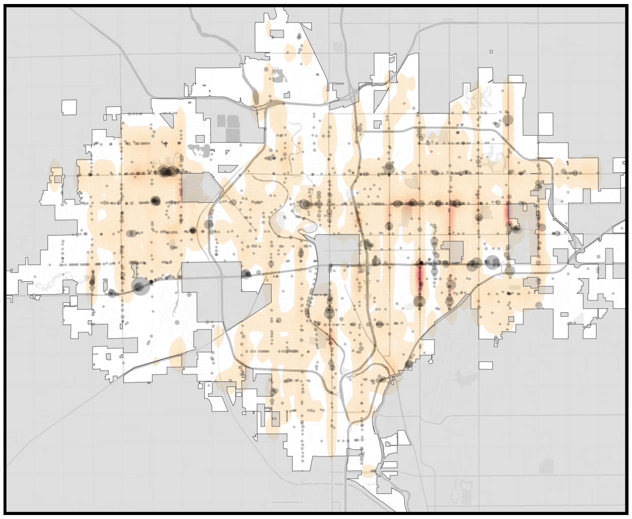

(b) Speeding violations and stops.

Fig. S11. A comparison of Wichita, KS's demographic composition, the location of speeding events, as measured by telematics data, and the location of speeding stops. (S11a): The demographics of residents, where red points represent non-Hispanic white residents and blue points represent non-white residents. (S11b): The density of speeding stops recorded by police officers, indicated by gray dots, where the area of the points is proportional to the number of stops at a single location; stops for speeding are overlayed with a heatmap of speeding events, as estimated from telematics data.

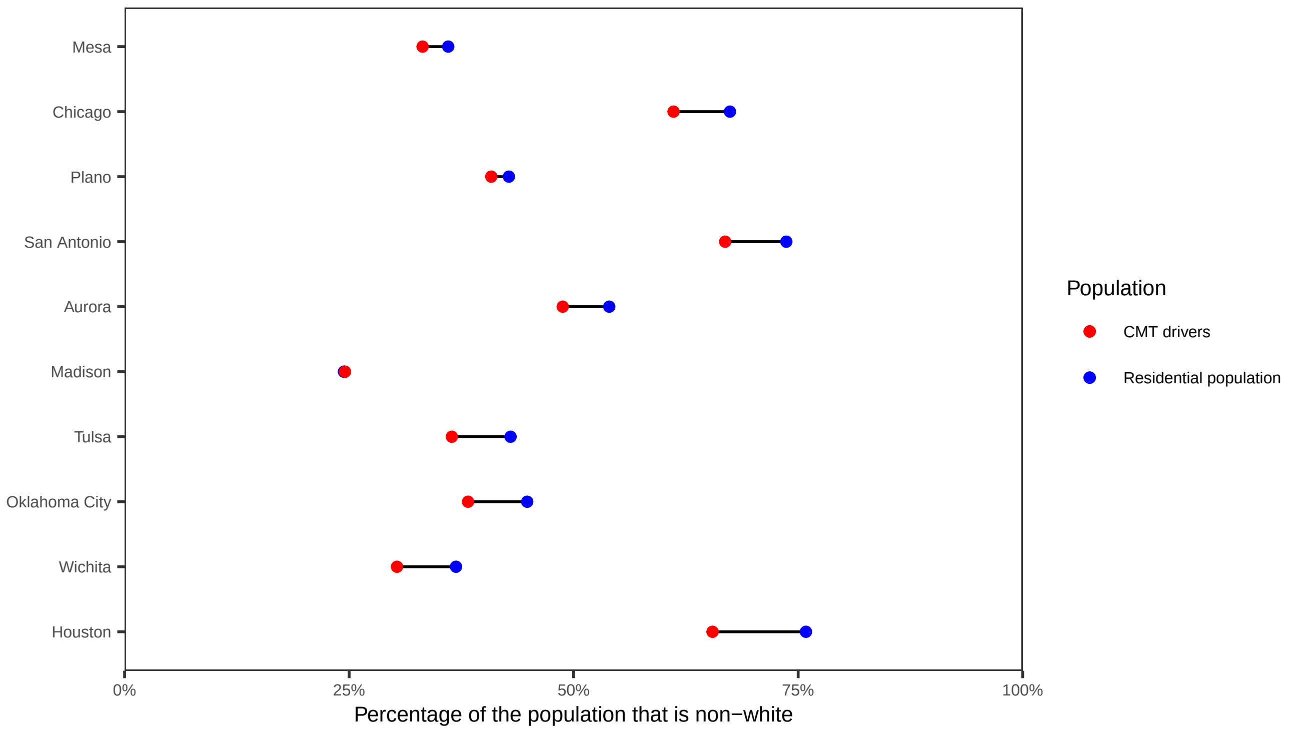

Fig. S12. Average demographic composition of police beats in each city, weighted by the beat's total residential population (blue) and the number of CMT drivers resident in the beat (red). That is, the blue dots represent $\left[\sum_i p_i \cdot m_i\right] / \left[\sum_i p_i\right]$ and the red dots $\left[\sum_i c_i \cdot m_i\right] / \left[\sum_i c_i\right]$, where p_i represents the number of residents of beat i, m_i the proportion of residents of beat i that are non-white, and c_i the number of CMT drivers resident in beat i. The average difference is 5%, and the maximum difference is 10%, in Houston.

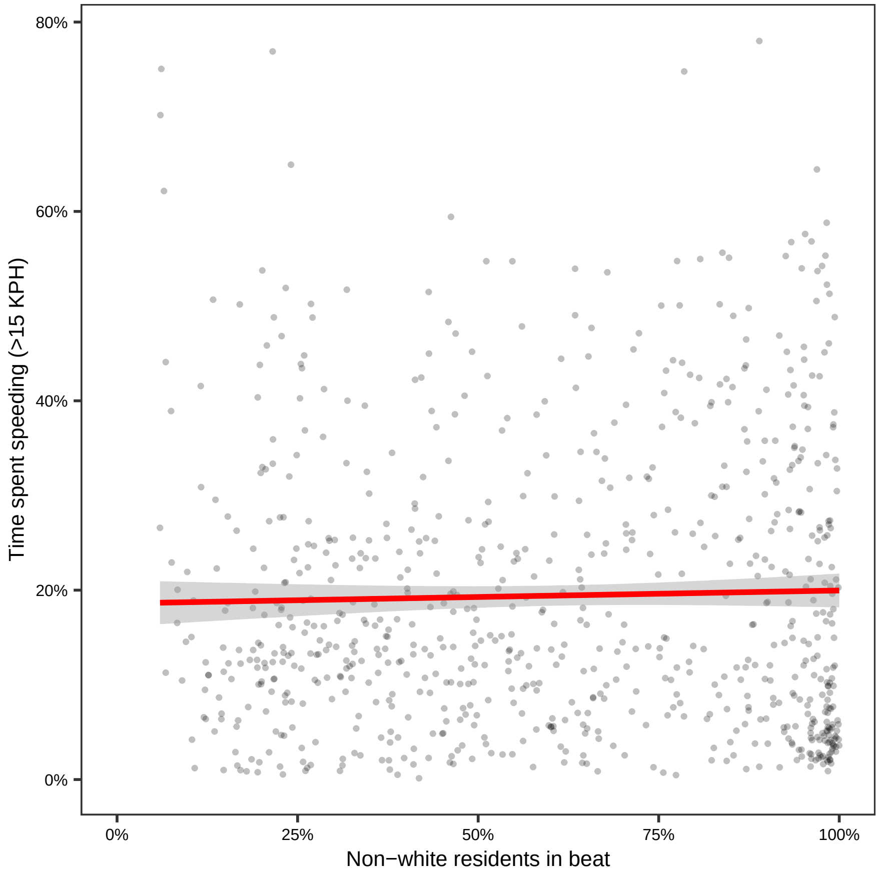

Fig. S13. The proportion of driving time spent at least 15 KPH above the speed limit according to TomTom data, versus the proportion of non-white residents, where each point represents a police beat in the 10 cities we analyze. The flat line of best fit (in red) indicates that neighborhood composition and speeding behavior are largely unrelated ($r = 0.03$). (Cf. Fig. 2.)

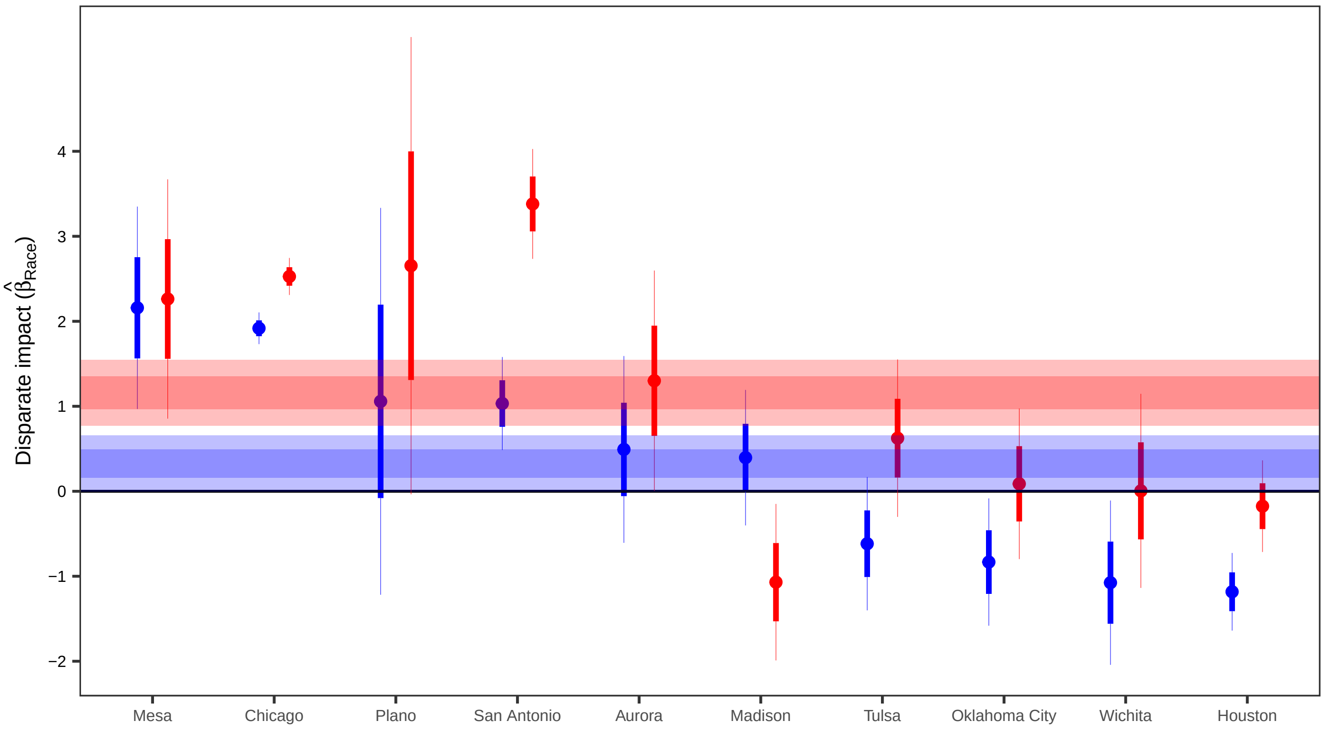

Fig. S14. Comparison of estimates for β_{RACE} in the main analysis when using CMT (blue) and TomTom (Red), as the data source. In general the estimates using TomTom suggest higher amounts of unjustified disparate impact, with the caveat that the TomTom data cover a shorter time period and cannot be post-stratified.

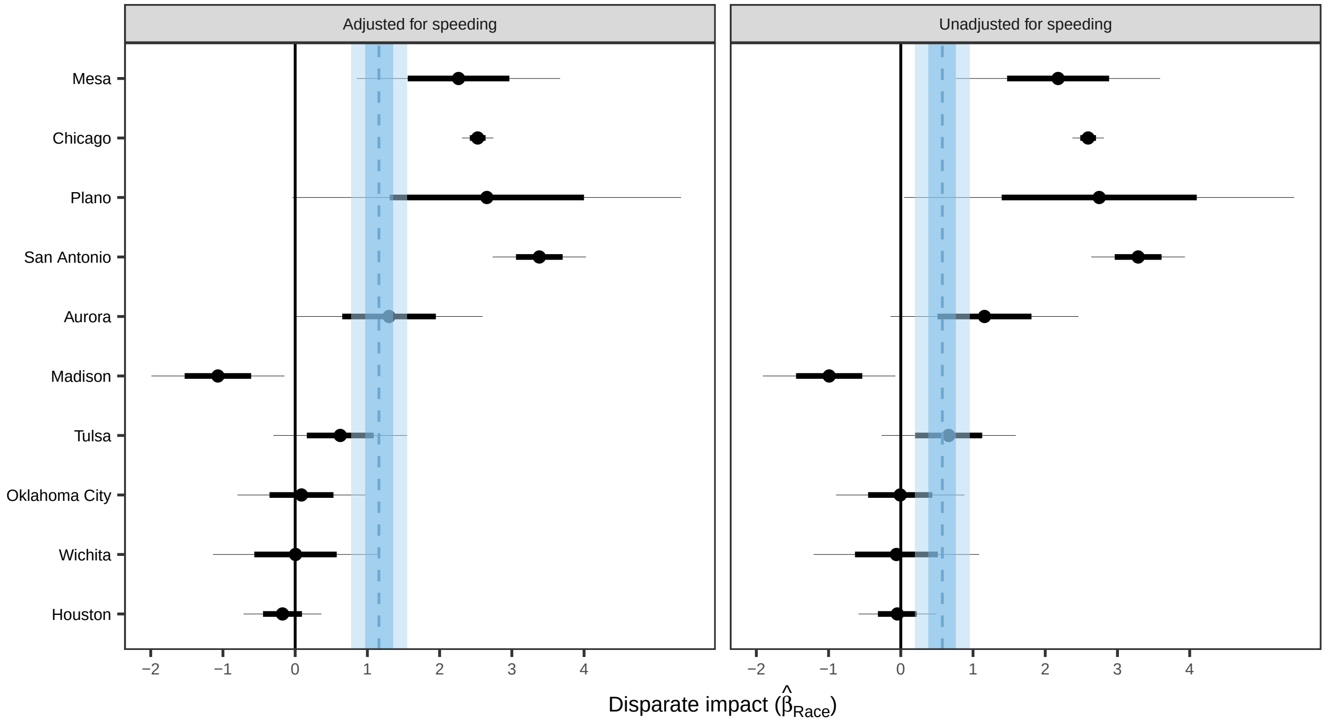

Fig. S15. The value of $\hat{\beta}_{\text{Race}}$ in our regression for each city using TomTom data, adjusting for the true prevalence of speeding (left) versus raw, unadjusted results (right). In both the adjusted and unadjusted regressions, we find significant heterogeneity across cities, suggesting that the nature and degree of policing practices varies considerably between cities. The mean of the unadjusted city-level coefficients is 0.58 (SE: 0.18, $p = 0.002$), meaning that speeding enforcement tends to be higher in areas with greater proportions of non-white residents. After adjusting for speeding using TomTom data, our average city-level coefficient is 1.16 (SE: 0.19, $p < 0.001$), meaning that disparities in speeding enforcement grow after adjusting for driving behavior. (Cf. Fig. 3, with the caveat that the TomTom data cover a shorter time period and cannot be post-stratified.)

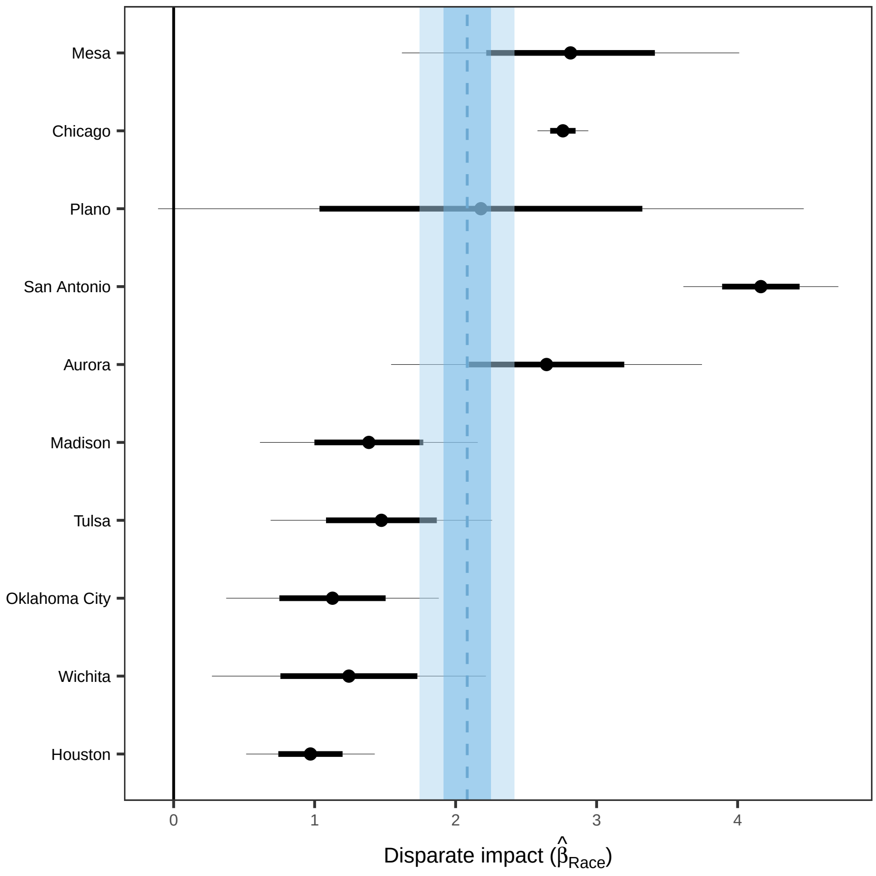

Fig. S16. The city-level values of $\hat{\beta}_{\text{Race}}$ in our regression for all traffic violations using TomTom data. We find uniformly positive coefficients with average 2.08 (SE: 0.17, $p < 0.001$), meaning that in every city we analyze, there were more recorded traffic violations in beats with higher proportions of non-white residents when not adjusting for true prevalence of violations. (Cf. Fig. 6, with the caveat that the TomTom data cover a shorter time period and cannot be post-stratified.)

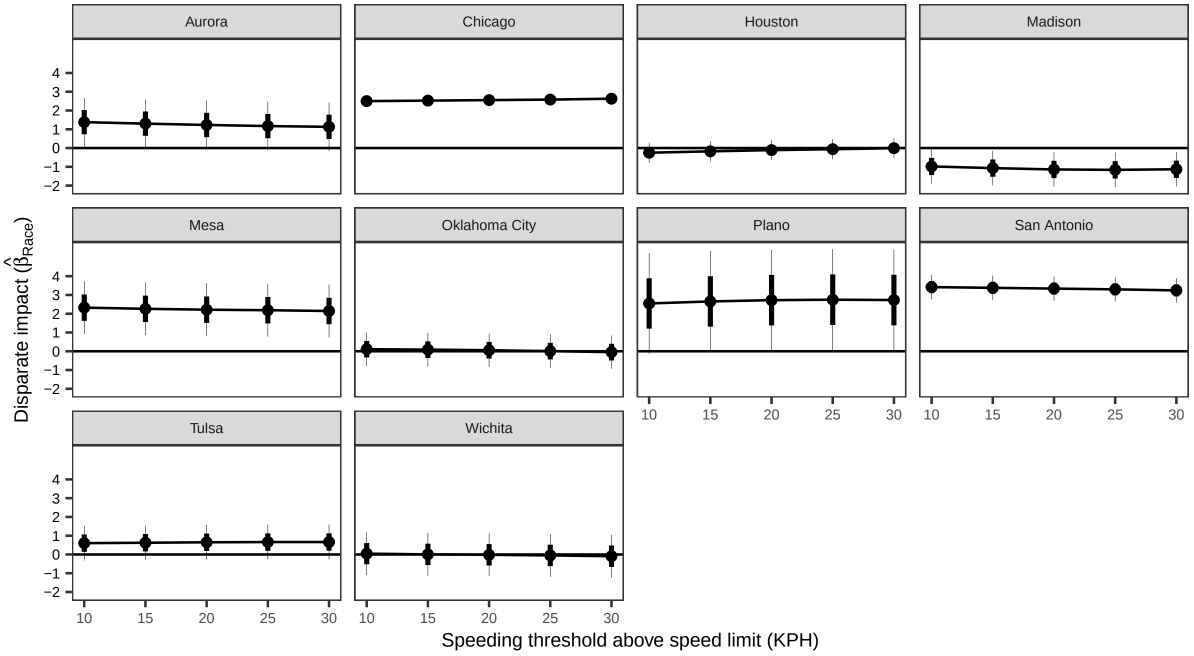

(a) Absolute speeding thresholds

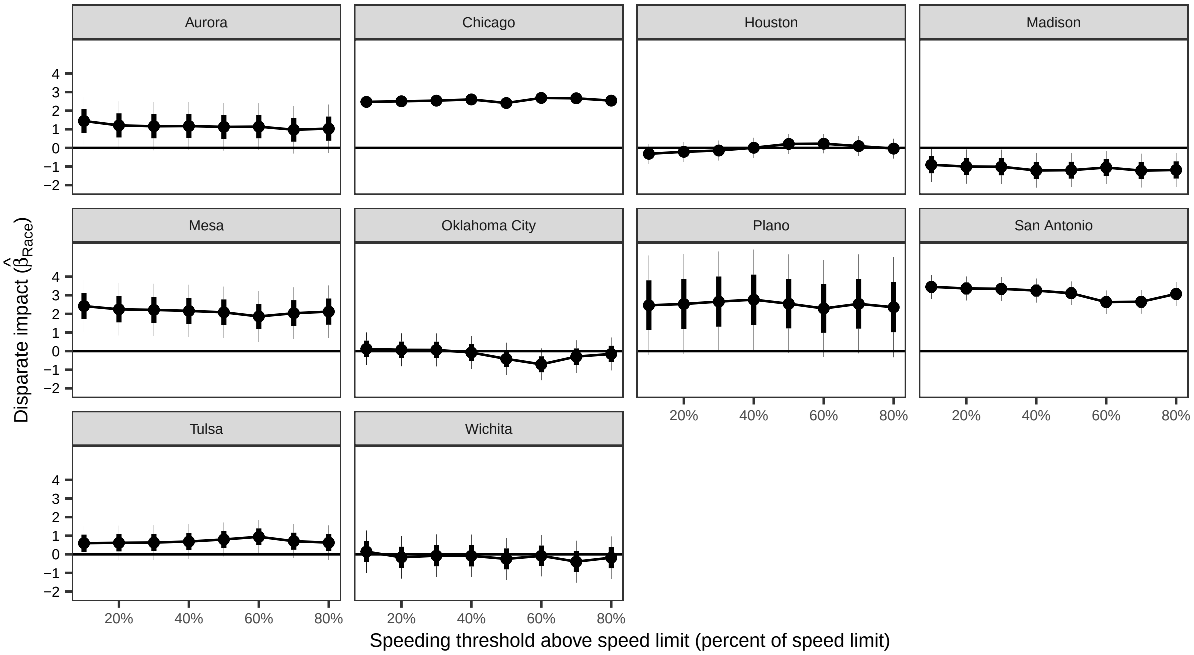

(b) Relative speeding thresholds

Fig. S17. Robustness of our main analysis to the definition of speeding using TomTom data. We show, for each city, the value of β_{RACE} as we vary the threshold above the speed limit we consider speeding when we compute d_i, on an absolute (S17a) and relative (S17b) scale. We find that our estimates of the coefficients are largely stable as we shift the threshold. (Cf. Fig. S1, with the caveat that the TomTom data cover a shorter time period and cannot be post-stratified.)

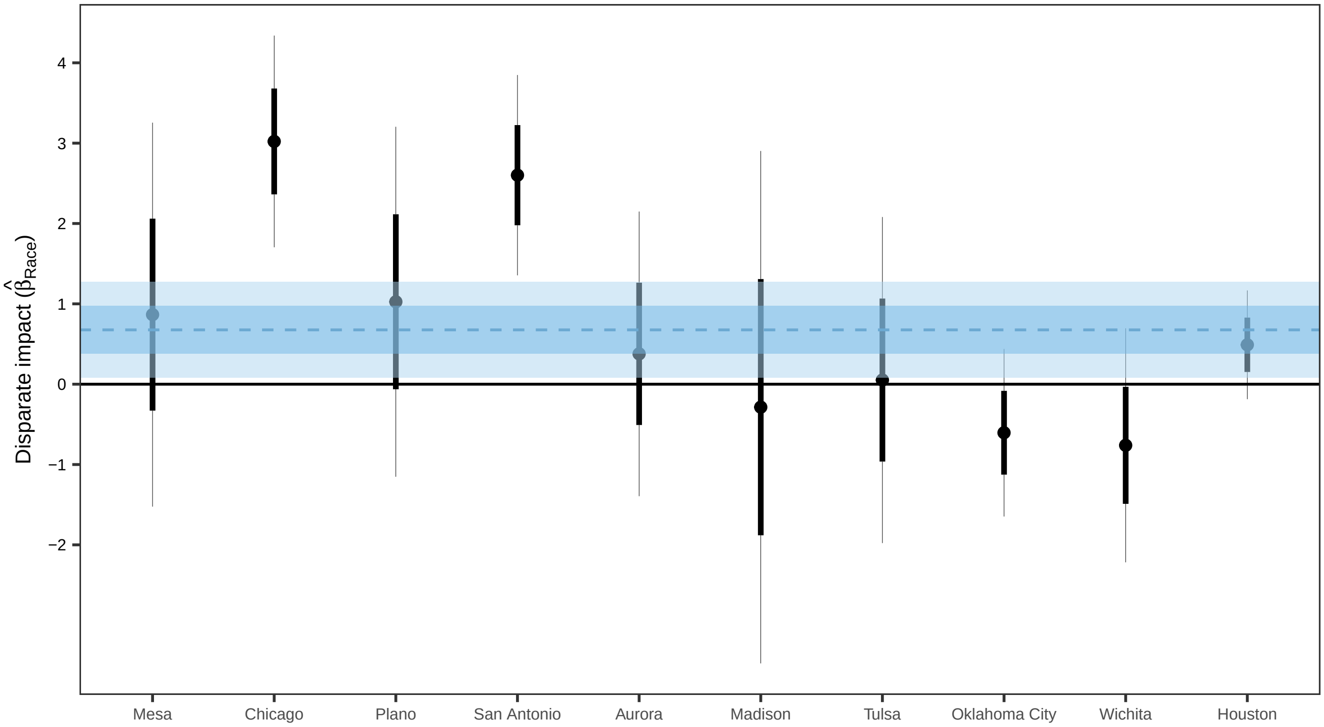

Fig. S18. Estimates of β_{RACE} in our speeding violation analysis using quasipoisson instead of negative binomial regression and TomTom data instead of CMT data. We find that the coefficients of Madison, Mesa, and Plano are all no longer statistcally significant in this specification. (Cf. Fig. S2, with the caveat that the TomTom data cover a shorter time period and cannot be post-stratified.)
